# Measurement invariance of a general cognitive performance measure across 27 European countries and Israel

**DOI:** 10.1007/s10433-025-00872-y

**Published:** 2025-07-29

**Authors:** Adrián García-Mollá, Irene Fernández, Amparo Oliver, José M. Tomás, Mireia Abella

**Affiliations:** Department of Methodology for the Behavioral Sciences, University of Valencia, Blasco Ibáñez Avenue, 21, 46010 Valencia, Spain

**Keywords:** Cognition, Measurement invariance, Cross-country comparisons, Older adults, SHARE

## Abstract

The use of global or composite cognitive measures is extended in both clinical and academic settings. In this line, several population-based surveys include measures of cognition that have frequently been combined into a single score. However, some methodological aspects of this practice have gone unnoticed. One of such aspects has been to provide evidence of the measurement invariance of the combined measure across countries involved in the surveys. Using data from the Survey of Health, Ageing and Retirement in Europe (SHARE), this study aims at providing evidence of the factor structure of a Global Cognitive Performance (GCP) measure and testing whether this structure remains invariant across 27 European countries and Israel. The sample was composed of 55,569 adults aged between 60 and 102 years old (*M* = 72.07, *SD* = 7.97). 56.58% were female. Confirmatory Factor Analysis was used to establish the measurement model of GCP in the general sample and within each country. Afterwards, measurement invariance across countries was evaluated using the traditional as well as the alignment approach. The unidimensional model of GCP deemed an adequate fit to the data in the general sample as well as within each country, except for Malta, which was excluded from further analyses. After dismissing full measurement invariance, we studied approximate measurement invariance using alignment. 31.85% of factor loading estimates were noninvariant, while 54.81% of item intercept estimates showed deviations from invariance. Given evidence of items’ intercepts and factor loadings noninvariance, researchers working with SHARE data should abstain from making cross-country comparisons of GCP. Some plausible explanations for noninvariance of items’ intercepts are further discussed.

**Supplementary Information:**

The online version contains supplementary material available at 10.1007/s10433-025-00872-y.

## Introduction

Populations are aging worldwide, due to increments in life expectancy accompanied by diminished birth rates (WHO 2024). However, the rise of life expectancy has taken place at a faster rate than increments in healthy life expectancy. This implies that, during the later years of life, individuals’ health status worsens with the onset of different diseases or disorders that diminish quality of life and make it difficult to lead an autonomous life (Salomon et al. 2012). Specifically, cognitive impairment in older people has become a major health issue (Wu et al. 2024). Different stages of cognitive ageing have been identified: normative impairment, Mild Cognitive Impairment (MCI) and dementia. More specifically, the latter two stages of cognitive impairment have shown major implications for the quality of life of older people affecting daily functioning (Johansson et al. 2015), promoting the occurrence of mood disorders (Ma 2020) and damaging the health status of the sufferer and their caregivers (Chapman et al. 2006).

There are different ways to operationalize cognition, but one common approach is assigning domains of functioning to specific brain regions (Harvey 2019). Cognitive domains have been well established in scientific literature through studies in which specific brain regions were lesioned and the impaired functions were studied (Babcock 1930; Damasio & Damasio 1989). An alternative method was the detection of certain functional impairments, and the posterior identification of the affected areas using neuroimaging techniques or by postmortem observation (Adolphs 2016; Vaidya et al. 2019).

In this line, several studies on the factorial validity of cognitive functioning measures showed better fit indices in multifactorial models than in unifactorial ones (Duro et al. 2009; Freitas et al. 2011; Siedlecki et al. 2010; Van Rentergem et al. 2020). Although the design of the Montreal Cognitive Assessment (MoCA) (Nasreddine et al. 2005) is multifactorial, its use to obtain a total score is quite widespread (Freitas et al. 2011). However, as reported in Duro et al., (2009), the two-factor structure, which classifies items into memory and attention/executive function, showed a better fit to the data than the unifactorial structure. In addition, the factor meta-analysis carried out by Van Rentergem et al. (2020) established that the best-fitting model of cognitive function, including different measures, was a multifactorial one.

Despite the multidimensional structure of cognitive functioning, in both clinical and academic settings, global scores of cognitive performance are usually employed. For example, Tsoi et al. (2015) reported the Mini-Mental State Examination (MMSE) (Folstein et al. 1975), which provides a global score of cognitive functioning, to be the most frequently used screening scale to diagnose dementia. However, this instrument has also been employed in research to provide a score of global cognition. For example, Choe et al. (2022) studied the relationship between serum copper levels and cognition, measured using the global MMSE score, in older adults. In the same line, Kim et al. (2014) examined the relationship between frailty and the Korean version of the MMSE. Similarly, Hao and Kim (2024) investigated the relationship between leisure activities and MMSE scores, considering the moderating effect of depression. In its part, the MoCA has been also employed as a global measure of cognition, in spite of its theoretical differentiation into cognitive domains. For instance, Chen et al. (2022) investigated the relationship between sedentary behavior and global, as well as domain-specific, MoCA scores. The use of such scales in the general older population is discouraged as ceiling effects have been reported (Spencer et al. 2013).

Still, it is common practice to combine scores from different subscales into an overall cognitive functioning score. In this vein, the Harmonized Cognitive Assessment Protocol (HCAP) (Langa et al. 2019) is a global study of cognitive aging that has been carried out in several countries including the United States of America, China, England, India, Mexico and South Africa. This protocol contains measures from several well-known instruments assessing cognition, such as the MMSE, the Consortium to Establish a Registry for Alzheimer’s Disease (CERAD) battery (Morris et al. 1989), or the Wechsler Memory Scale (Wechsler 1987). Researchers have combined these measures to create specific cognitive domains, but they also report a global cognitive score or dimension (Gross et al. 2020, 2023; Jones et al. 2024; Liu et al. 2025). However, the use of such global scores of cognition entails a certain loss of specificity in the assessment of specific domains (Floden et al. 2015; Johnson et al. 2007). As an example, Wood et al. (2020) reported the total score of the MoCA test to have limitations in making differential diagnoses between Alzheimer’s Disease (AD) and primary progressive aphasia dementia. Nevertheless, when examining the subscales, these authors observed significant differences between the two groups. A plausible explanation for this is that the most noticeable symptom in early stages of AD concerns memory impairment, while in primary progressive aphasia dementia early symptoms manifest as language difficulties (Kirshner 2012). Furthermore, given its extended use in the detection of dementia, different studies have employed the MMSE for the detection of MCI (Conde-Sala et al. 2012; Modrego & Gazulla 2013; Palmqvist et al. 2012); however, it has been shown to lack sensitivity for detecting early stages of cognitive impairment (Arevalo-Rodriguez et al. 2021).

There are different longitudinal surveys in the field of aging that include cognitive performance measures such as the Health and Retirement Study (HRS) (Sonnega et al. 2014), the English Longitudinal Study of Ageing (ELSA) (Banks et al. 2024), the China Health and Retirement Report (CHARLS) (Zhao et al. 2012), or the Survey of Health, Ageing and Retirement in Europe (SHARE) (Börsch-Supan et al. 2013). Specifically in SHARE, the most used cognitive measures include the 10-word recall test (Harris & Dowson 1982) for recent and delayed recall, a verbal fluency score (Ardila et al. 2006), a numeracy score based on subtractions and a temporal orientation score. The two latter tests are based on items from the Mini-Mental State Examination test (Folstein et al. 1975).

As far as we know, validity of the global score of cognitive function using the measures included in SHARE has not been studied. Be that as it may, and in line with the aforementioned literature, studies using SHARE data have employed different combinations of them to form a global score of cognition (Cermakova et al. 2018; Han et al. 2021; Nielsen et al. 2022; Portellano-Ortiz and Conde-Sala 2018). For example, Cermakova et al. (2018) and Han et al. (2021) employed recent and delayed recall together with verbal fluency to create a global score. In turn, Nielsen et al. (2022) used all four measures, as did Portellano-Ortiz and Conde-Sala (2018).

Furthermore, scales providing a general score of cognitive performance can also face issues with cross-cultural validity, given that a certain instrument may be optimal for a group of individuals but not for others, due to cultural or educational differences (Siedlecki et al. 2010; Vissoci et al. 2019). As an example, the systematic review conducted by Steis and Schrauf (2009) shows that the translation and adaptation of the Mini-Mental State Examination into 15 different languages presents comparability issues across cultures, which could be attributed to contextual and cultural differences. As argued by Ardila (2020), it is crucial to keep cultural differences in mind when assessing cognitive performance since there are documented differences in learning processes and contextual experiences, such as the frequency of word use or the presence of certain elements in the context.

In this study, we aim at testing a unidimensional factor structure of global cognition among older adults using available cognitive scores from SHARE data. Namely, performance in word recall, verbal fluency, orientation and numeracy will be employed as items of global cognition. After establishment of the factor structure, we will further assess cross-country measurement invariance. First, we will try to replicate the same model in each country individually. Then, we will test measurement invariance using a traditional invariance routine as well as the alignment approach.

## Materials and methods

### Sample and procedure

Data employed in this study comes from the most recent wave (9th) of the Survey of Health, Ageing and Retirement in Europe (SHARE-ERIC 2024; Börsch-Supan et al. 2013). SHARE is a longitudinal panel study that collects data about physical and mental health, social networks, retirement and income from individuals aged 50 years old or older and their partners, irrespective of their age. As this study was aimed at establishing a measurement model of cognition for older adults, we only selected those individuals who were aged 60 years or older at the time of the interview. Data collection of 9th wave took place between 2021 and 2022.

The sample included a total of 55,569 participants aged between 60 and 102 years old, and a total of 28 countries were represented in this wave: Austria (4.6%), Germany (6%), Sweden (3.9%), Netherlands (3.6%), Spain (2.7%), Italy (5.7%), France (4.3%), Denmark (3.4%), Greece (4.8%), Switzerland (3%), Belgium (6%), Israel (1.2%), Czech Republic (5.2%), Poland (6.9%), Luxembourg (1.3%), Hungary (2,6%), Portugal (1.9%), Slovenia (5.8%), Estonia (6.1%), Croatia (6.4%), Lithuania (2%), Bulgaria (1.3%), Cyprus (1.1%), Finland (2.7%), Latvia (2.4%), Malta (1.4%), Romania (2.2%) and Slovakia (1.4%). Sociodemographic characteristics and descriptive statistics of employed variables are presented in Table 1.

**Table 1:** Means, percentages, standard deviations, minimum and maximum of the variables in the study

	Median (SD) or %
Age	71 (7.97)
Gender	
Male	43.2%
Female	56.8%
ISCED 1997	
Code 0. Pre-primary education	2.6%
Code 1. Primary education or first stage of basic education	13.2%
Code 2. Lower secondary education or second stage of basic education	16.7%
Code 3. Upper secondary education	38.8%
Code 4. Post-secondary non-tertiary education	4.8%
Code 5. First stage of tertiary education	22.7%
Code 6. Second level of tertiary education	0.9%
Other	0.2%
Temporal orientation	
0	0.6%
1	0.7%
2	1.8%
3	9.1%
4	87.9%
Numeracy	
0	1.4%
1	5.6%
2	4.7%
3	9.5%
4	15.1%
5	63.7%
Recent recall	5 (1.76)
Delayed recall	4 (2.13)
Verbal Fluency	20. (7.93)

SD = Standard deviation; Min. = Minimum; Max. = Maximum; ISCED (*International Standard Classification of Education*

### Instruments

The global cognitive factor was operationalized by means of recent and delayed recall, temporal orientation, numeracy and verbal fluency.

Memory was measured using the 10-word recall test (Harris & Dowson 1982). Participants were read a randomly assigned one of four lists of ten words and they were asked to recall them two times: immediately after having the list read (recent recall) and after a delayed time (delayed recall). Both items have a 0 to 10 range scale, and scores indicate the number of correctly recalled words.

Numeracy represents the mathematical performance of respondents but also implies working memory and concentration. It consists of a series of five subtractions. At each attempt, the individual is asked to subtract 7 from the present number, starting at 100. The serial seven test is part of the MMSE (Folstein et al. 1975) and the score ranges between 0 and 5, indicating the number of correct subtractions. The test was corrected so that errors were not cumulative, e.g. if the first subtraction was missed but the second was correct, a correct answer was counted.

Verbal fluency was measured by asking the participants to report as many animals as possible in one minute (Ardila et al. 2006). It is a test that involves executive function, language and semantic memory (Rosen 1980).

Temporal orientation included four items measuring the respondents’ orientation to date, month, year and day of week. Every correct response adds 1 point, so the variable ranges from 0 to 4. This item is also based on the MMSE (Folstein et al. 1975).

### Statistical analysis

First, the one-factor model of General Cognitive Performance (GCP) was tested using Confirmatory Factor Analysis (CFA). After, we tested the same model in each country separately. Once this model’s fit to the data was deemed adequate both at the general level and for each country individually, we followed a traditional measurement invariance routine (Jöreskog 1971): first, we tested an unconstrained configural model; then, we tested a metric invariance model by constraining factor loadings to be equal across countries; finally, we tested a scalar invariance model with constrained factor loadings and item intercepts across countries. Finally, we employed free and fixed alignment for approximate measurement invariance.

Regarding this last procedure, the alignment method overcomes some of the limitations of the traditional approach with large-scale surveys (Byrne and van de Vijver 2017). Namely, these authors note the traditional procedure to be too restrictive, yielding substantial parameter misspecification. Moreover, Byrne and van de Vijver (2017) also point out the limited functionality of SEM programs, that compare one group with all other groups at a time, which implies respecifying the same model a substantively high number of times to assess partial measurement invariance when the number of groups is high.

The alignment method entails a three-step sequence. First, a configural model is established, which then goes through an optimization process in order to minimize measurement non-invariance, which yields estimation of the parameters. Finally, further tests are computed on the estimates to compare their approximate invariance across groups. To establish the reference group, a free alignment was first estimated. After assessing the country with the lowest factor mean, fixed alignment was employed by constricting that country’s factor mean to zero. A cut-off point of 25% of noninvariant parameters was established as the criterion to assess the integrity of latent mean estimates (Muthén and Asparouhov 2014).

The estimation method employed in this study was Robust Maximum Likelihood (MLR). Fit was addressed with the mostly used and most recommended statistics and indexes (Kline 2016): the chi-square statistic (χ^2^), the Comparative Fit Index (CFI), the Standardized Root Mean Square Residual (SRMR), and the Root Mean Squared Error of Approximation (RMSEA). Acceptable model fit was considered in the presence of CFI values equal or greater than 0.90 and SRMR/RMSEA values lower than 0.08, as established by Marsh et al. (2004). For the traditional invariance routine, we employed Cheung and Resvold’s (2002) criterion of CFI differences (ΔCFI) of constrained against less constrained models under 0.01 as indicating no substantial deterioration, favoring the constrained, more parsimonious, model. We assessed fit of the alignment method using each item’s fitting functions for the intercept and factor loading, which represent each parameter’s contribution to the final simplicity function (Byrne and van de Vijver 2017). Lower item’s overall contribution to the fitting function is considered an indicator of item noninvariance. Moreover, R^2^ was employed to measure each parameter’s degree of invariance, with larger values indicating greater parameter invariance (Asparouhov and Muthén, 2014). Missing data was handled using Full Information Maximum Likelihood (FIML), that estimates each parameter using the value that maximizes the likelihood function based on all available data for each parameter (Enders 2010). Analyses were done in Mplus 8.11 (Muthén & Muthén, 2017). MPlus syntax is available in the Supplementary Material.

## Results

### Confirmatory factor analysis

**Fig. 1:**
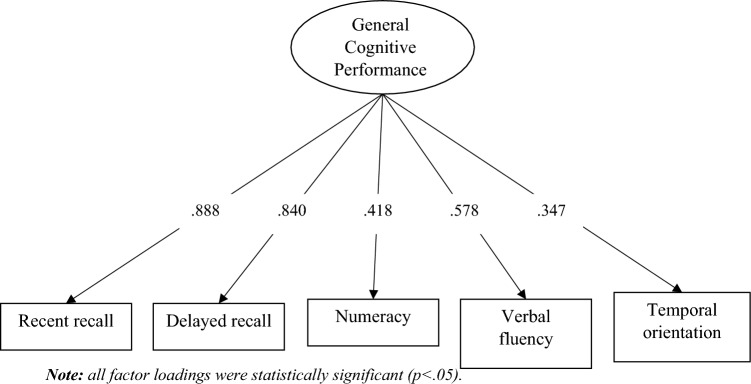
Standardized factor loadings of the CFA model in the overall sample

The one-factor model of GCP was estimated and tested, and standardized loadings are presented in Fig. 1. Fit indexes showed adequate model fit to the data: χ^2^(5) = 2011.86, *p* < 0.001, CFI = 0.967, SRMR = 0.040, RMSEA = 0.085, [90% CI 0.082–0.088]. After establishment of the factor structure in the general sample, we estimated the model in each country separately. Model fit results are available in Table 2. Taking into account the limitations of RMSEA with relatively few degrees of freedom (Kenny et al. 2015), the only country in which CFI was clearly inadequate was Malta. We decided to exclude this country from the following analyses, as this result shows that the unidimensional structure of GCP does not fit the data from Maltese participants.

**Table 2:** Model fit results of the unidimensional model of GCP in each country (country code between brackets)

Country	χ^2^	df	p	CFI	RMSEA	90% CI RMSEA	SRMR
Austria (11)	79.77	5	< 0.001	0.976	0.076	0.062–0.092	0.046
Germany (12)	63.32	5	< 0.001	0.976	0.059	0.047–0.073	0.031
Sweden (13)	82.78	5	< 0.001	0.966	0.084	0.069–0.101	0.048
Netherlands (14)	33.34	5	< 0.001	0.986	0.054	0.037–0.072	0.026
Spain (15)	83.70	5	< 0.001	0.950	0.102	0.083–0.121	0.046
Italy (16)	172.43	5	< 0.001	0.948	0.103	0.09–0.116	0.045
France (17)	73.67	5	< 0.001	0.975	0.075	0.061–0.091	0.033
Denmark (18)	69.72	5	< 0.001	0.970	0.083	0.066–0.101	0.044
Greece (19)	219.79	5	< 0.001	0.949	0.127	0.113–0.141	0.071
Switzerland (20)	23.56	5	< 0.001	0.989	0.047	0.029–0.067	0.026
Belgium (23)	71.94	5	< 0.001	0.983	0.063	0.051–0.077	0.033
Israel (25)	13.78	5	< 0.001	0.991	0.052	0.020–0.086	0.033
Czech Republic (28)	82.14	5	< 0.001	0.970	0.073	0.059–0.087	0.036
Poland (29)	109.68	5	< 0.001	0.965	0.074	0.062–0.087	0.036
Luxembourg (31)	33.95	5	< 0.001	0.969	0.089	0.062–0.119	0.048
Hungary (32)	39.29	5	< 0.001	0.972	0.069	0.050–0.090	0.041
Portugal (33)	38.79	5	< 0.001	0.978	0.080	0.058–0.104	0.035
Slovenia (34)	190.42	5	< 0.001	0.957	0.107	0.094–0.120	0.046
Estonia (35)	149.41	5	< 0.001	0.960	0.092	0.080–0.105	0.043
Croatia (47)	197.86	5	< 0.001	0.963	0.105	0.092–0.117	0.042
Lithuania (48)	98.79	5	< 0.001	0.940	0.128	0.107–0.151	0.053
Bulgaria (51)	34.52	5	< 0.001	0.969	0.092	0.065–0.122	0.036
Cyprus (53)	31.13	5	< 0.001	0.942	0.092	0.063–0.124	0.057
Finland (55)	30.36	5	< 0.001	0.985	0.058	0.039–0.079	0.025
Latvia (57)	127.67	5	< 0.001	0.929	0.136	0.116–0.157	0.058
Malta (59)	81.49	5	< 0.001	0.840	0.140	0.115–0.168	0.069
Romania (61)	69.53	5	< 0.001	0.933	0.103	0.082–0.125	0.061
Slovakia (63)	58.87	5	< 0.001	0.962	0.117	0.091–0.145	0.047

### Measurement invariance

We first followed the traditional invariance routine to compare the unidimensional structure of GCP across all countries except Malta. Results of the configural, metric and scalar models are available in Table 3. Results show that a common factor structure of GCP holds across the 26 European countries and Israel. However, when constraining factor loadings to be equal across countries (*i.e.* metric invariance), CFI differences indicate substantial model deterioration. This situation becomes completely evident when further constraining item intercepts to be equal across countries (*i.e.* scalar invariance).

**Table 3:** Model fit results of the traditional invariance routine

Model	χ^2^	df	*p*	CFI	ΔCFI	RMSEA	90% CI RMSEA	SRMR
Configural	2239.41	135	< 0.001	0.964	–	0.088	0.085–0.091	0.043
Metric	3649.53	239	< 0.001	0.942	0.022	0.084	0.082–0.086	0.105
Scalar	9779.17	343	< 0.001	0.840	0.124	0.116	0.115–0.118	0.150

Given the obvious lack of invariance across countries and the limited functionality of SEM software to test partial measurement invariance, we employed the alignment method to study approximate measurement invariance across the 26 European countries and Israel. We employed free alignment to estimate latent factor means of the countries and determine the country with the lowest factor mean, that would be established as the reference group. Austria has the latent factor mean closest to 0 and hence used as the reference group.

After estimation of the free model, we computed fixed alignment with Austria as the reference group, constraining its factor mean to zero. Given five variables (recent recall, delayed recall, numeracy, verbal fluency and temporal orientation) and 27 groups, we found a 31.85% of noninvariant factor loadings (43 of a total of 135), indicating low noninvariance. With regard to the intercepts, 54.81% of the parameters were noninvariant (74 of a total of 135), which suggests that intercepts’ degree of noninvariance is substantially high. Approximate measurement invariance per country is presented in Table 4. Results showed parameter noninvariance in terms of both item intercepts and factor loadings.

**Table 4:** Approximate measurement invariance (noninvariance) of GCP

Indicator	Country
Item intercepts	
Recent recall	11 12 (13) (14) 15 16 17 (18) 19 (20) (23) 25 28 (29) (31) 32 33 34 35 47 48 51 53 55 57 61 63
Delayed recall	11 12 (13) (14) 15 16 (17) (18) 19 (20) (23) 25 28 (29) 31 32 33 (34) (35) (47) 48 51 53 55 (57) 61 63
Numeracy	(11) (12) (13) (14) (15) 16 17 (18) 19 (20) (23) 25 (28) (29) (31) (32) (33) (34) (35) (47) 48 (51) (53) 55 57 61 (63)
Verbal fluency	(11) (12) (13) (14) (15) (16) (17) (18) (19) 20 (23) 25 (28) (29) (31) (32) (33) (34) (35) (47) (48) 51 (53) (55) 57 (61) 63
Temporal Orientation	11 12 13 14 (15) (16) 17 18 (19) 20 23 25 28 (29) 31 (32) (33) (34) (35) (47) (48) (51) (53) 55 (57) (61) (63)
Factor loadings	
Recent recall	11 12 13 14 15 16 17 18 (19) 20 23 25 28 29 31 32 33 34 35 (47) 48 51 55 57 61 (63)
Delayed recall	11 12 13 14 (15) (16) 17 18 19 20 23 (25) 28 (29) 31 32 (33) (34) (35) (47) (48) (51) (53) 55 57 (61) (63)
Numeracy	11 12 13 14 (15) (16) (17) 18 19 20 23 25 28 (29) 31 (32) (33) (34) (35) (47) (48) (51) 53 55 (57) (61) 63
Verbal fluency	11 12 13 14 (15) (16) 17 (18) (19) 20 23 25 28 29 (31) 32 (33) 34 35 47 (48) 51 (53) 55 57 (61) 63
Temporal Orientation	11 12 13 14 (15) (16) 17 18 19 20 23 25 28 29 31 32 33 34 35 47 (48) 51 53 55 57 (61) (63)

Countries codes are: 11 = Austria, 12 = Germany, 13 = Sweden, 14 = Netherlands, 15 = Spain, 16 = Italy, 17 = France, 18 = Denmark, 19 = Greece, 20 = Switzerland, 23 = Belgium, 25 = Israel, 28 = Czech Republic, 29 = Poland, 31 = Luxembourg, 32 = Hungary, 33 = Portugal, 34 = Slovenia, 35 = Estonia, 47 = Croatia, 48 = Lithuania, 51 = Bulgaria, 53 = Cyprus, 55 = Finland, 57 = Latvia, 61 = Romania, 63 = Slovakia. Brackets indicate country did not display invariance of the parameter

Information regarding the fitting function for items’ intercepts and factor loadings, and R^2^ is displayed in Table 5. Regarding items’ contribution to fitting function, we observe that recent recall shows the lowest overall contribution to the fitting function (− 249.71) and therefore can be considered the item with the least amount of noninvariance. The recent recall item is also the one displaying the largest R^2^ value, whose higher values imply a higher degrees of parameter invariance. Standardized parameter estimates of factor loadings and item intercepts for each country are available in Table S1 from the Supplementary Material for further scrutiny of differences across countries.

**Table 5:** Alignment fit statistics of GPC across countries

Item	Factor loadings		Item intercepts		Loadings + Intercepts
	Fit function contribution	R^2^	Fit function contribution	R^2^	Total contribution
Recent recall	− 124.38	0.357	− 125.33	0.933	− 249.71
Delayed recall	− 145.35	0.000	− 135.58	0.939	− 280.94
Numeracy	− 219.41	0.172	− 211.78	0.541	− 431.19
Verbal fluency	− 143.49	0.326	− 247.89	0.507	− 391.38
Temporal orientation	− 190.48	0.213	− 211.89	0.041	− 402.37

Estimated latent factor means of each country are available in Table 6 in decreasing order. Next to each country’s latent factor mean, Table 6 also displays the countries with statistically significant lower estimated values (*p* < 0.05). Results from Table 6 show Luxembourg to be the country displaying the highest estimated factor mean of GCP. Notably, there seems to be a tendency for northern and central countries to present higher estimated factor means, while southern and eastern European countries rank at the bottom regarding their estimated latent factor mean.

**Table 6:** Latent factor mean comparisons of GCP across countries

Rank	Country	Mean value	Countries with significantly smaller factor mean (*p* < 0.05)
1	Luxembourg (31)	0.202	11 20 18 32 13 12 28 14 23 55 17 35 19 34 57 51 25 16 61 29 63 47 53 48 15 33
2	Austria (11)	0.000	13 12 28 14 23 55 17 35 19 34 57 51 25 16 61 29 63 47 53 48 15 33
3	Switzerland (20)	− 0.011	13 12 28 14 23 55 17 35 19 34 57 51 25 16 61 29 63 47 53 48 15 33
4	Denmark (18)	− 0.019	13 12 28 14 23 55 17 35 19 34 57 51 25 16 61 29 63 47 53 48 15 33
5	Hungary (32)	− 0.025	13 12 28 14 23 55 17 35 19 34 57 51 25 16 61 29 63 47 53 48 15 33
6	Sweden (13)	− 0.136	55 17 35 19 34 57 51 25 16 61 29 63 47 53 48 15 33
7	Germany (12)	− 0.146	55 17 35 19 34 57 51 25 16 61 29 63 47 53 48 15 33
8	Czech Republic (28)	− 0.157	55 17 35 19 34 57 51 25 16 61 29 63 47 53 48 15 33
9	Netherlands (14)	− 0.161	55 17 35 19 34 57 51 25 16 61 29 63 47 53 48 15 33
10	Belgium (23)	− 0.172	55 17 35 19 34 57 51 25 16 61 29 63 47 53 48 15 33
11	Finland (55)	− 0.260	19 34 57 51 25 16 61 29 63 47 53 48 15 33
12	France (17)	− 0.289	19 34 57 51 25 16 61 29 63 47 53 48 15 33
13	Estonia (35)	− 0.311	19 34 57 51 25 16 61 29 63 47 53 48 15 33
14	Greece (19)	− 0.463	51 25 16 61 29 63 47 53 48 15 33
15	Slovenia (34)	− 0.474	51 25 16 61 29 63 47 53 48 15 33
16	Latvia (57)	− 0.530	25 16 61 29 63 47 53 48 15 33
17	Bulgaria (51)	− 0.633	47 48 15 33
18	Israel (25)	− 0.676	15 33
19	Italy (16)	− 0.678	29 47 48 15 33
20	Romania (61)	− 0.733	15 33
21	Poland (29)	− 0.734	15 33
22	Slovakia (63)	− 0.741	15 33
23	Croatia (47)	− 0.745	15 33
24	Cyprus (53)	− 0.748	15 33
25	Lithuania (48)	− 0.799	15 33
26	Spain (15)	− 1.066	
27	Portugal (33)	− 1.090	

## Discussion

A considerable body of academic literature employs a global measure of cognitive functioning based on items from different domains. Nevertheless, the use of this type of scales or global scores may lead to loss of specificity since the information from several domains is summarized into a single global score (Li et al. 2023; Mayer et al. 2002). As far as we know, this is the first study to examine the structural validity and the measurement invariance of this extended global measure of older adults’ cognition across SHARE participating European countries and Israel. First, we followed the traditional measurement invariance routine (Jöreskog 1971). However, this procedure has been deemed impractical when the number of groups to be compared is large and a researcher wants to examine partial measurement invariance (Byrne and van de Vijver 2017). Thus, we followed the proposal set forth by Muthén and Asparouhov (2014), and employed the maximum likelihood alignment approach, given that it is argued to overcome the limitations of the traditional measurement invariance routine.

In general terms, our findings suggest that the measurement of GCP presents several differences across countries. Results from the unidimensional GCP model suggested an adequate fit to the data both in the general sample and within each country separately, with the exception of Malta. The value of the RMSEA was slightly over the standards. However, previous research has already noted this tendency in simple models with relatively few degrees of freedom (Kenny et al. 2015). In such cases, Shi et al. (2020) suggest evaluating model fit using CFI and SRMR instead. Taking this into consideration, we concluded that the fit of the unidimensional GCP model could be deemed adequate in general and country-specific samples except for Malta. Thus, we excluded Malta of subsequent measurement invariance analyses. This finding implies that the measurement model of GCP does not represent the data from Maltese participants; therefore, SHARE users and researchers should avoid scores based on this GCP factor structure with Maltese respondents.

After establishment of the measurement model, we tested whether this structure held constant across the remaining 26 European countries and Israel. Initially, a standard routine testing constrained against less constrained configural, metric and scalar models was adopted, following the traditional procedure (Jöreskog 1971). Results from this routine showed serious flaws of invariance. Moreover, this procedure entails a series of restrictive assumptions and limitations when trying to assess partial measurement invariance that makes it impractical when applied to a large number of groups (Byrne & van de Vijver 2017).

Therefore, we employed the alignment approach to examine approximate measurement invariance. First, we used the free alignment method to establish a country as the reference group. Next, we tested measurement invariance across countries using the fixed alignment method. Results showed that the recent recall item presents the least degree of noninvariance. This result could be explained by the fact that recent recall is usually unaffected in the general population. Short-term memory detriment is widely employed as an indicator of severe conditions (Möllers et al. 2022; Zokaei et al. 2020). Moreover, long-term memory is considered a more complex domain than short-term memory, given that the former depends on the latter (Čepukaitytė et al. 2023). This entails that delayed recall items could have more power to discriminate among degrees of impairment, while recent recall scores may be most useful on the detection of serious cases.

The evidence of substantial item intercepts’ non-invariance suggests that cross-country comparisons of GCP scores based on the reported measurement model should be made, if at all, with extreme caution (Jöreskog 1971; Wicherts & Dolan 2010). As deviations from metric invariance are substantially smaller, relationships between GCP and other relevant variables might be compared with prudence across countries (Byrne and van de Vijver 2017). Be that as it may, GCP scores could still be employed at the general sample level, although the adequacy of these scores for different aims such as predicting, detecting or assessing change needs further study given previous evidence (Floden et al. 2015; Johnson et al. 2007).

Results also suggest northern and central countries to exhibit higher estimated means of GCP, whereas southern and eastern countries ranked lower. Given its important role in the prevention of cognitive impairment, these regional differences could be attributed to education and economy (Perez and Matsaganis 2021). As exposed by Betthäuser et al. (2021), not only should educational rankings be taken into account, but also inequality of educational opportunities. In this respect, income might be defining the educational opportunities insofar as it has been seen that there is an abrupt difference between the countries of southern and eastern regions with respect to those of northern and central areas (Boltho 2020). These results could be in line with Niu et al. (2017), who found a higher prevalence of dementia in southern and eastern European countries compared to northern and central Europe. However, as item intercepts were not invariant across groups, latent factor means reported in this study ought to be interpreted with extreme caution.

Another plausible explanation for differences across countries could be due to cultural and linguistic differences. Neuropsychological scales have been shown to display issues in their adaptation to other languages and cultures (Gibbons et al. 2002; Vissoci et al. 2019). For example, results by Siedlecki et al. (2010) showed partial non-invariance of item intercepts of a neuropsychological battery employed for the assessment cognition between Spanish and English speakers. As exposed by Ardila (2020), the development of cognitive function throughout the lifecycle and the subsequent impairment in older adults implies many environmental and cultural factors that are not taken into account in most standardized measures. As shown by our results, several differences among countries in items’ intercepts were found. This result suggests that the same item could be more difficult in some countries than in others. In addition, the factor loadings also showed a high degree of noninvariance among the 28 countries. This result implies that items are not explained equivalently by GCP in every country. Therefore, future research should abstain from using a GCP score in cross-country comparisons.

Although this study highlights the inadequate performance of a global measure of cognition across SHARE participating countries, it also has certain limitations that need to be acknowledged. The first one is related to data gathering. The complex data collection process in population-based studies, such as SHARE, involves a large number of variables of different areas being measured in a short period of time (Börsch-Supan et al. 2013). Thus, measures of cognitive functioning may be coarse and uninformative. In this respect, two of the cognitive measures include in SHARE (orientation and numeracy) are adapted from the MMSE, whose use in general populations has been shown to provide skewed distributions of responses that lead to ceiling effects (Spencer et al. 2013). In this study, we tried to deal with this issue by employing a robust estimation method. Nonetheless, non-normality could have resulted in model misfit and failures of invariance, which could have influenced the results (Kline 2016). The second limitation refers to the cross-cultural functioning of GCP. Given the complex nature of cognitive impairment and its development throughout the lifecycle, identifying which factors determine the differential functioning of cognitive functioning measures constitutes a major challenge (Meitinger et al. 2020; Wicherts 2016).

## Conclusions

This study reports evidence of differential functioning of a GCP measure among 28 countries participating in SHARE. Not only does this overall measure vary significantly across countries due to the different factor loadings, but there are also differences in the intercepts. This suggests that the items imply more difficulty in responding in some countries than in others. In this vein, future research must be oriented to identify the factors that explain the substantial differences in relation to cultural or environmental traits.

## Supplementary Information

Below is the link to the electronic supplementary material.

Additional file1.

## Data Availability

The data that support the findings of this study are available from the SHARE Project but restrictions apply to the availability of these data, which were used under license for the current study, and so are not publicly available. Data are however available from the authors upon reasonable request and with permission of the SHARE Project.
